# Improving motivation and treatment uptake behaviors of patients with eating disorders using patient narrative videos: study protocol of a pilot randomized controlled trial

**DOI:** 10.1186/s40337-023-00960-3

**Published:** 2024-01-02

**Authors:** Melissa-Claire Daugelat, Joachim Kimmerle, Daniela Hagmann, Kathrin Schag, Katrin Elisabeth Giel

**Affiliations:** 1 Department of Psychosomatic Medicine and Psychotherapy, Medical University Hospital Tübingen, Osianderstr. 5, 72076 Tübingen, Germany; 2 Centre of Excellence for Eating Disorders Tübingen (KOMET), University of Tübingen, Tübingen, Germany; 3 Leibniz-Institut für Wissensmedien, Tübingen, Germany; 4 Department of Psychology, Eberhard Karls University Tübingen, Tübingen, Germany; 5 Child and Adolescent Psychiatry, Medical University Hospital Tübingen, Tübingen, Germany; 6 DZPG (German Center for Mental Health), Tübingen, Germany

**Keywords:** Eating disorders, Motivation, Help-seeking, Lived experience, Patient narratives

## Abstract

**Background:**

Patients with eating disorders (ED) typically report delays between the onset of symptoms and engagement with treatment services. Personal barriers including stigma, shame, and guilt, as well as the availability of social support may influence patients’ decisions to engage with treatment services. Patient narratives are personalized stories discussing the illness and recovery of previously affected persons. Such narratives can reduce self-stigma and provide current patients with hope for their own recovery.

**Method:**

This pilot study will examine the effects of patient narrative videos on the treatment motivation and uptake of treatment services for patients with ED. Three narrative videos were developed from the perspectives of (a) a former patient with an ED, (b) an ED specialist, and (c) the same former patient discussing a somatic condition unrelated to ED. Patients will be randomized into three video viewing and one treatment-as-usual group. Effects on treatment motivation will be assessed using the University of Rhode Island Change Assessment Scale (URICA-S) immediately after viewing the videos, as well as one-week and three-month follow-ups. Treatment uptake will be assessed during follow-up using a questionnaire listing possible treatment interactions. A post-intervention questionnaire and semi-structured interviews will be used to assess the feasibility and acceptability of patient narrative videos for this population.

**Discussion:**

There is an urgent need to encourage patients with ED to engage with specialized treatments as soon as possible. Patient narratives may be a pivotal approach to implementing cost effective and easy to disseminate early intervention programs to future patients with ED.

## Public significance statement

Currently, there exists an urgent need to close the treatment gap and encourage patients with eating disorders (ED) to engage with specialized treatment services as soon as possible. Narrative videos featuring the experiences and perspectives of former patients could be a pivotal approach to implementing cost effective and easy to disseminate early interventions to patients currently affected by ED.

## Introduction

Treatment for eating disorders (ED) has been shown to be most effective when initiated shortly after the onset of symptoms [1]. Nevertheless, patients typically report a significant delay between the onset of ED symptoms and their engagement with treatment services. The duration of untreated ED (DUED), i.e., the time between onset of the ED and first contact with treatment, ranges from 2.5 years for Anorexia Nervosa to 6 years for Binge-Eating-Disorder [2]. Longer DUED is associated with a progression of ED symptoms, increased psychological distress, and increased mortality among patients with Anorexia [2, 3]. Patients with ED often report long wait times and/or a lack of specialized treatment services as contributing to the DUED they experienced [4, 5]. Interventions such as the First Episode Rapid Early Intervention for Eating Disorders (FREED) service model in the UK aim to reduce these healthcare-related delays by optimizing the referral process for tailored and evidence-based ED treatment services [6]. Meanwhile, feelings of ambivalence or a lack of motivation from patients to change their behaviors have also been reported as contributing to the DUED of most EDs [7]. In fact, many patients will wait up to 12 months after recognizing their ED symptoms before deciding to seek treatment [7]. The Transtheoretical Model of Health Behavior Change (TTM) by Prochaska and Di Clemente [8] can be used to understand patients’ motivation (or lack thereof) to recover from their ED. The TTM proposes that when changing a health behavior, an individual will gradually progress through six stages of willingness to change, namely precontemplation, contemplation, preparation, action, maintenance, and termination. Evidence suggests that patients who have a higher motivation to change prior to commencing treatment (i.e., are further along the stages of change) will demonstrate better treatment outcomes, including reduced dietary restriction, binging, and purging behaviors [9, 10].

Engagement with treatment services may be influenced by the social support patients can rely upon [11, 12]. In fact, the presence [13, 14] or absence [15, 16] of social support may either facilitate or impede a patient’s decision to seek and/or engage with treatment services. Unfortunately, a common theme among individuals with ED is a general lack of social connections outside the immediate family, with many individuals utilizing websites and online forums in the hopes of finding support, acceptance, and a place of belonging among fellow ED patients [17]. Currently, there is an increased use of peers in the care of various mental disorders, including ED (e.g., [18, 19]). Peer mentorship programs with former and currently recovered ED patients have shown high engagement rates, as well as increased mood, quality of life, and hope for a possible recovery [20]. Similarly, information shared by fellow ED patients has been reported by patients to be significantly more empowering than when the same information was presented by healthcare professionals [21].

Patient narratives, i.e., personalized stories of persons previously affected by a mental disorder detailing their illness and recovery, provide another promising adjunct to ED treatments. Patient narratives are available both as published books and in a variety of formats throughout the internet [22, 23]. Studies have shown that reading patient narratives may reduce the self-stigma associated with ED in student populations [24] and, when presented in a safe and responsible way, may shorten the duration of DUED [25] and provide current patients with feelings of hope for a possible recovery [22]. However, to date only a handful of studies have investigated the effects of patient narratives in ED care [26]. Studies on other severe mental disorders, such as psychosis (e.g., [27, 28]), suggest that viewing patient narrative videos can increase self-efficacy and provide hope toward recovery. These effects were strongest after viewing videos featuring former patients, in comparison to videos featuring healthcare professionals, despite the presentation of identical recovery-oriented content [27]. Patients with psychosis typically report similar levels of ambivalence toward treatment as patients with ED [29], and these findings suggest that perceptions of similarity/authenticity allow former patients with lived experience to inspire hope for recovery among persons currently affected by the disorder [27]. Three further clinical trials are currently being completed as part of the Narrative Experiences Online (NEON) Study [30], in which the effects of patient narrative videos are being examined for persons experiencing psychosis and other mental health problems. To the best of our knowledge, no prior studies have examined the effects of evidence-based and recovery-focused patient narratives presented as videos for patients with ED.

## Study aims

The proposed study will examine the effects of patient narratives provided in video format for patients with ED. In particular, the role of patient narrative videos on patients’ treatment motivation and uptake of treatment services will be assessed. The study further aims to ascertain the feasibility and acceptability of patient narratives, as well as establishing data to identify suitable outcomes and sample size calculations for a larger trial.

Patients will be randomly assigned to view one of three narrative videos, presented from the perspective of a former and currently recovered patient with an ED (Video A), an ED specialist (Video B), and the same former patient reporting on a somatic condition unrelated to ED (Video C). It is expected that patients who receive access to narrative videos from the perspective of a former patient with an ED (Video A) or an ED specialist (Video B) will show:

- stronger increases in treatment motivation (Hypothesis 1).
- and increased likelihood to engage in treatment uptake behaviors (Hypothesis 2).

compared to patients who receive access to a narrative video unrelated to ED (Video C) or patients who receive access to no videos.

It is further expected that patients who receive access to a narrative video from the perspective of a former patient with an ED (Video A) will show:

- stronger increases in treatment motivation (Hypothesis 3).
- and increased likelihood to engage in treatment uptake behaviors (Hypothesis 4).

compared to patients who receive access to a narrative video from the perspective of an ED specialist (Video B).

## Method/design

This study protocol is reported according to the recommendations of the SPIRIT checklist [31].

### Study participants

Female patients with EDs who attend a consultation appointment at the outpatient services of the Department of Psychosomatic Medicine and Psychotherapy at the Medical University Hospital Tübingen will be informed about the study and invited to participate. During such consultation appointments patients complete a variety of diagnostic assessments and are provided with recommendations for treatment. Patients do not receive any counselling or treatment at these appointments. Patients will be asked to provide information about any previous treatment(s) they may have received and will be excluded if their last treatment occurred within the last four weeks prior to the start of the study. As the majority of patients begin experiencing EDs already in late adolescence, patients aged 15 years and older will also be recruited from the Child and Adolescent Psychiatry at the Medical University Hospital Tübingen. Patients with any full-syndrome or subclinical ED diagnosis will be invited to participate in this study to gain a better understanding of any transdiagnostic differences that may affect for whom this intervention is best suited. In order to further increase reach and diversity, the study will also be made available to individuals who seek obesity treatment at the Medical University Hospital Tübingen, provided they also display symptoms of an ED.

### Inclusion criteria

Patients must comply with all of the following criteria in order to be eligible for the trial:

- Full-syndrome or subclinical ED according to DSM-5 (diagnosed by a trained clinician).
- Minimum age 15 years.
- Female gender.
- Written informed consent.

### Exclusion criteria

- Acute suicidality.
- Psychotic disorders (lifetime).
- Bipolar disorder (lifetime).
- Current moderate-severe substance use disorders.
- Currently receiving ED treatment(s).

### Materials used

#### Self-report measures

Demographic and diagnostic information, including ED diagnosis, will be obtained from patient files. Patients will be provided with a definition of patient narratives before being asked if/what experiences they may have had with patient narratives, and in what format. The following questionnaires will be used to determine ED psychopathology, comorbid mental disorders, treatment motivation, previous and current treatment uptake behaviors, and hope and confidence toward recovery:

- ED psychopathology will be assessed using the Eating Disorders Examination Questionnaire (EDE-Q [32]). The EDE-Q consists of 28 items measuring the cognitions and eating disturbances associated with ED, as well as diagnostically relevant behavioral features of ED. Items can be allocated to four subscales: Restraint, Eating Concern, Shape Concern, and Weight Concern. Mean subscale scores and a mean global score are calculated in order to determine overall eating pathology [32]. The EDE-Q has high internal consistency (α ≤ 0.97) and is sensitive to change [33].
- Comorbid mental disorders will be assessed using the Patient Health Questionnaire (PHQ-D [34]). The PHQ-D consists of 15 items and is used to screen for symptoms of depressive disorders, panic disorder, and psychosocial functioning. The PHQ-D has excellent criterion validity and good internal consistency for depressive disorders (α = 0.88) [34].
- Treatment motivation will be assessed using the University of Rhode Island Change Assessment Scale—Short Version (URICA-S [35]). The URICA-S consists of 16 items, measuring four of the six stages of change (i.e., Pre-contemplation, Contemplation, Action, and Maintenance) according to the TTM [8]. Readiness for change scores will be calculated for each patient by summing the mean contemplation, action, and maintenance subscale values of the URICA-S, before subtracting the mean precontemplation subscale value [36]. The URICA-S shows acceptable to good internal consistency (α = 0.61–0.85), as well as strong criterion validity [35].
- Treatment uptake behaviors will be assessed using a short questionnaire developed for this study. Patients will be asked to select from a list which treatment services they have previously or currently engaged with, and to what extent (e.g., phone contact, appointment made/attended). ED treatment services are varied, including inpatient, partial inpatient, and outpatient treatment in psychiatric/psychosomatic clinics, outpatient psychotherapeutic and nutritionist services, and digital eHealth interventions. For the purposes of this study, treatment uptake will be defined as attending appointments at any of these treatment providers, however, preparatory actions such as researching treatment options and time spent on the wait list will also be assessed.
- Hope and confidence toward recovery will be assessed using two visual analogue scales. Patients will be asked to indicate how hopeful and confident they are regarding their own recovery. Each patient’s scores will be calculated by measuring the distance (0-100 mm) on the visual analogue scales between the anchor (no hope/confidence) and the patient’s selection, with higher scores indicating greater hopefulness and confidence toward recovery.
- A post-intervention questionnaire will be used to assess patients’ feedback regarding the authenticity, empathy, and likeability of the person featured in the narrative video, as well as the usefulness of the video(s) as a whole. A subgroup of six patients (determined through Research Randomizer [37]) will also be invited to participate in a semi-structured interview to provide further insights into their perception of the authenticity, empathy, likeability, and usefulness of the video(s), so as to allow for a more thorough and qualitative understanding of how these are experienced by patients.

#### Interventions

The narrative videos used in this intervention were developed collaboratively in a participatory research process with a former patient with an ED, while also being based on the results of a recent systematic review of barriers and facilitators for help-seeking in patients with ED [12]. Preliminary versions of these videos will be trialed in a pre-test study with a healthy student population. The final videos will be approx. six minutes in length (+/- 1 min difference) and feature a person of the same age and gender. The former patient featured in the video is a young woman with a healthy body weight, and it is not explicitly stated which symptoms or ED diagnosis she suffered from. Instead, EDs are discussed as a whole, and the content of the videos focuses on specific aspects of the former patient’s treatment journey, including what motivated her to seek treatment and how her life has changed post-recovery. As part of the pilot study presented here, a parallel 4-arm pilot randomized controlled trial (RCT) approach will be used to evaluate the efficacy of patient narrative videos. Patients will be randomized into one of four groups for the intervention: one group (IG1) will receive access to a video featuring the personal experiences of a former patient with an ED regarding the barriers and facilitators she faced when seeking treatment, with particular focus on stigma, shame, and guilt, as well as the importance of social support. The second group (IG2) will receive access to a video featuring an ED specialist, in which the same barriers and facilitators to treatment presented by the recovered patient will be discussed, but from a professional perspective. The Control Group (CG) will receive access to a video in which the recovered patient will discuss the same barriers and facilitators to treatment, but for a somatic condition unrelated to ED (i.e., torn knee ligament). The remaining patients randomized in the Treatment-as-Usual Group (TAU) will not receive access to any videos.

### Procedure

**Fig. 1:**
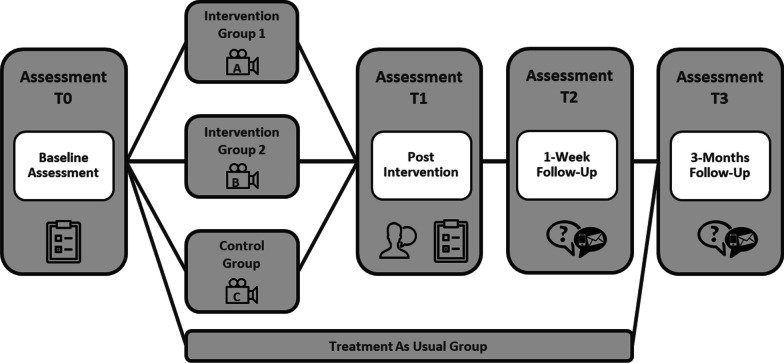
Study plan

**Fig. 2:**
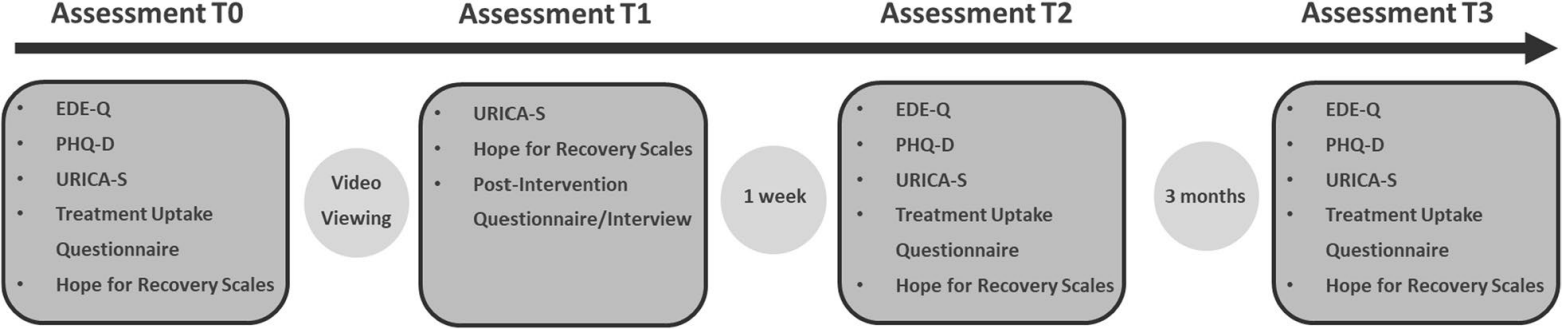
Overview of assessment materials

An overview of the study plan and assessment materials are presented in Figs. 1 and 2, respectively. Following a baseline assessment (T0), patients will receive access to one of the three videos in a controlled setting at the Medical University Hospital Tübingen. After watching the videos (T1), patients will complete a series of questionnaires to assess any immediate changes in treatment motivation. A subgroup of patients will also be invited to participate in a semi-structured interview. Patients will be contacted for follow-up one week (T2) and three months (T3) after watching the videos. TAU patients will complete assessments at T0 and T3 but will not receive access to any videos.

### Outcomes

#### Main outcome measures

Main outcome measures of this study will be changes in patients’ treatment motivation and treatment uptake behaviors. Changes in treatment motivation will be measured by comparing patients’ baseline (T0) readiness for change scores (URICA-S) to scores immediately after viewing the videos (T1), at one-week (T2) and three months follow-up (T3). Changes in treatment uptake will be measured by comparing the amount of treatment seeking activities indicated at baseline (T0), at one-week (T2) and three months follow-up (T3).

#### Secondary ED-related outcome measures

Additional outcome measures will include changes in ED psychopathology, as measured by changes in the scores of the EDE-Q, and changes in patients’ hopefulness and confidence for their own recovery, as measured by increased values in the visual analogue scales immediately after viewing the videos (T1) at one-week (T2) and three months follow-up (T3).

#### Evaluation of narrative videos

The suitability of the narrative videos will be determined through a post-intervention questionnaire and qualitative interviews at T1, in which patients will be asked their views on the authenticity, empathy, and likeability of the person featured in the video, as well at the usefulness of the video(s) as a whole.

### Sample size

Currently, there is limited prior evidence on the effects of patient narratives on the treatment motivation of patients with ED. Existing studies are primarily qualitative in design (e.g., [22]). Further, no studies have previously used video narratives to examine their effect on treatment motivation. The proposed study is a pilot project and as such, its main aims are to investigate the usefulness of the narratives, and to identify a meaningful primary outcome and establish data to allow for sample size calculations for a larger trial. Analogous to similar pilot trials, it is planned to include 25 patients in each study group, resulting in a sample size of 100 patients. Dropout cases will be replaced until the proposed sample size has been recruited.

### Randomization/blinding

Patients will be randomized into one of four groups for the intervention. The study will be an open label study, meaning that neither the study team nor patients will be blind regarding group allocation. Randomization will occur following the receival of written consent to participate, using Research Randomizer [37].

### Statistical analyses

#### Treatment motivation

Generalized linear mixed effects models will be used to assess the relationship between patients’ group allocation (IG1/IG2/CG/TAU) and their readiness for change scores at T0, T1, T2, and T3.

#### Treatment uptake behaviors

Pearson’s chi-square tests of contingencies will be used to assess the relationship between patients’ group allocation (IG1/IG2/CG/TAU) and treatment uptake behaviors at T0, T2, and T3.

#### Hope for recovery

Generalized mixed effects models will be used to assess the relationship between patients’ group allocation (IG1/IG2/CG/TAU) and hope for recovery at T0, T2, and T3.

#### Evaluation of narrative videos

Pearson’s chi-square tests of contingencies will be used to assess the relationship between patients’ group allocation (IG1/IG2/CG) and their evaluation of the narrative videos at T1. Narrative/descriptive syntheses will also be completed, in which patients’ evaluations of the videos will be extracted from interview transcripts and coded under themes.

### Data management and data monitoring

As part of this study, personal data will be collected, stored, and analyzed. All self-report questionnaires will be completed by patients online via UniPark (www.unipark.com/de). The UniPark data centers in Germany meet the highest security requirements including certifications according to ISO/IEC 27,001 and SOC II, as well as compliance with the requirements of the GDPR. Participant codes will be assigned, i.e., data storage will be pseudonymized. No external data monitoring will be implemented as this is a pilot trial.

### Ethical aspects

#### Ethical approval

Ethical approval for completion of the trial has been obtained by the ethics committee of the Medical Faculty at the University Hospital Tübingen (reference 419/2022BO1). No known risks or negative side effects are expected to occur as a result of participation. Written informed consent will be obtained from all patients as well as the parents/guardians of patients under the age of 18. Patients may withdraw consent at any time, for any reason, without loss of benefit.

## Discussion

To date, a limited number of studies have examined the usefulness and influence of patient narratives in the recovery process of mental disorders in general [26], and even fewer studies did so for patients with ED. Among those studies who examined patients with ED (e.g., [22]), reading a selection of patient narratives, such as autobiographies by former patients, showed no measurable short-term effects on motivation or self-efficacy. Nevertheless, the majority of patients reported that reading patient narratives initiated feelings of hope regarding their own recovery [22]. Even less research has been completed regarding the use of patient narratives in an audio-visual format. To the best of our knowledge, this will be the first study to examine the effects of evidence-based and recovery-focused patient narratives presented as videos for patients with ED.

### Strengths and limitations

A strength of this study is the strong integration of lived experience into the intervention, which has been increasingly advocated [19, 38, 39]. A further strength is the inclusion of clinically relevant outcome measures, including treatment uptake behaviors at several time points in response to engagement with patient narratives. The inclusion of such measures will allow the results of this study to more easily translate into and inform clinical practice. The long follow-up period of three months is roughly in accordance with the average wait times for psychotherapeutic treatment in Germany [40, 41], although this varies depending on region, disorder, and type of service. The study design will allow both short-term and long-term effects of this intervention to be examined. Limitations of this study include a selection bias, as the recruitment focuses on patients attending a diagnostic appointment at the Medical University Hospital Tübingen. Nevertheless, a large proportion of persons who attend such appointments fail to undertake any further actions, and therefore may benefit greatly from this intervention.

## Conclusion

There is currently an urgent need to encourage and assist patients with ED to engage with specialized treatments as soon as possible. If effective, patient narratives may be a pivotal approach to implementing cost effective and easy to disseminate early intervention programs to future patients with ED.

## Data Availability

All data used to support the findings of this study are available from the corresponding author upon reasonable request.
